# Cytosolic phospholipase A_2_ (cPLA_2_) IVA as a potential signature molecule in cigarette smoke condensate induced pathologies in alveolar epithelial lineages

**DOI:** 10.1186/s12944-016-0300-x

**Published:** 2016-08-15

**Authors:** Subodh K. Yadav, Sanjeev K. Sharma, Abdullah Farooque, Gaurav Kaushik, Balwinder Kaur, Chander M. Pathak, Bilikere S. Dwarakanath, Krishan L. Khanduja

**Affiliations:** 1 Department of Biophysics, PGIMER Chandigarh, Chandigarh, 160012 India; 2 Institute of Nuclear Medicine and Allied Sciences, New Delhi, India; 3 Present address: Department of CSIC, PGIMER Chandigarh, Chandigarh, 160012 India; 4 Present address: Surgery, School of Medicine, KU Medical Center (KUMC), Kansas City, KS 66160 USA; 5 Present address: Central Research Facility, Sri Ramachandra University, Porur, Chennai, 600116 India

**Keywords:** Cigarette smoke condensate, Cytosolic phospholipases A_2_, Reactive oxygen species, Inflammation, A549 cells, WI26 cells

## Abstract

**Background:**

Smoking is one of the leading causes of millions of deaths worldwide. During cigarette smoking, most affected and highly exposed cells are the alveolar epithelium and generated oxidative stress in these cells leads to death and damage. Several studies suggested that oxidative stress causes membrane remodeling via Phospholipase A_2_s but in the case of cigarette smokers, mechanistically study is not yet fully defined. In view of present perspective, we evaluated the involvement of cytosolic phospholipase A_2_ (cPLA_2_) IVA as therapeutic target in cigarette smoke induced pathologies in transformed type I and type II alveolar epithelial cells.

**Methods:**

Transformed type I (WI26) and type II (A549) alveolar epithelial cells were used for the present study. Cigarette smoke condensate (CSC) was prepared from most commonly used cigarette (Gold Flake with filter) by the Indian population. CSC-induced molecular changes were evaluated through cell viability using MTT assay, reactive oxygen species (ROS) measurement using 2,7 dichlorodihydrofluorescin diacetate (DCFH-DA), cell membrane integrity using fluorescein diacetate (FDA) and ethidium bromide (EtBr) staining, super oxide dismutase (SOD) levels, cPLA_2_ activity and molecular involvement of specific cPLA_2_s at selected 24 h time period.

**Results:**

CSC-induced response on both type of epithelial cells shown significantly reduction in cell viability, declined membrane integrity, with differential escalation of ROS levels in the range of 1.5–15 folds and pointedly increased cPLA_2_ activity (*p* < 0.05). Likewise, we observed distinction antioxidant potential in these two types of lineages as type I cells had considerably higher SOD levels when compared to type II cells (*p* < 0.05). Further molecular expression of all cPLA_2_s increased significantly in a dose dependent manner, specifically cytosolic phospholipase A_2_ IVA with maximum manifestation of 3.8 folds. Interestingly, CSC-induced ROS levels and cPLA_2_s expression were relatively higher in A549 cells as compared to WI26 cells.

**Conclusions:**

The present study indicates that among all cPLA_2_s, specific cPLA_2_ IVA are the main enzymes involved in cigarette smoke induced anomalies in type I and type II lung epithelial cells and targeting them holds tremendous possibilities in cigarette smoke induced lung pathologies.

**Electronic supplementary material:**

The online version of this article (doi:10.1186/s12944-016-0300-x) contains supplementary material, which is available to authorized users.

## Background

Cigarette smoking is leading cause of deaths and projected to cause 8–10 million deaths per year worldwide [1]. It is associated with different types of lung cancer and approximately one third of all cancer death [1–3]. Currently more than 370 billion cigarettes are being consumed by smokers globally and it has been projected that more than 30 % of the people will be smokers by 2030 [2]. On the other hand it has been expected that rate of smoking will be reached 70 % in developing countries [1]. It has been documented that single puff of cigarette smoke contains 10^17^ oxidant molecules out of which 10^15^ are reactive oxygen species/ reactive nitrogen species. Moreover, these ROS/RNS are known to be one of the causative factors in various lung pathologies including cancer and chronic obstructive pulmonary disease (COPD) [4–6]. During cigarette smoking, the most affected and highly exposed cells are the alveolar epithelium which is lined by type-I (~90–95 %) and type-II (~5–10 %) epithelial cells. In addition, damage and death of epithelial cells induced by cigarette smoke exposure can mostly be accounted for by an increased in oxidant stress. Enhanced levels of free radicals/oxidants leads to oxidative stress and initation of repair processes whether started as part of an inflammatory response or as a response to injury is still not clear in cigarette smokers. In this context, one of the hallmark enzymes are Phospholipase A_2_s which are responsible for membranes remodeling [7–9] but in the case of cigarette smokers mechanistically study is not yet fully defined.

PLA_2_s are lipolytic enzymes that catalyze the hydrolysis of acyl-groups at the sn-2 position of glycerophospholipids and produce free fatty acids and lyso-Phospholipids by an interfacial activation catalytic mechanism. To date, at least 26 genes that encode various types of PLA_2_ proteins with esterase’s activity have been identified in human and are assigned to five different groups. (i) Secretory PLA_2_s with molecular weight of 14 kDa, (ii) the 85 kDa cytosolic PLA_2_s, (iii) Ca^2+^ independent PLA_2_s (iv) Platelet-activating factor acetyl hydrolase and (v) lysosomal PLA_2_s. These PLA_2_s were differentiated on the basis of their sequence, molecular weight, disulfide bonding patterns, requirement for Ca^2+^ to their biochemical characteristics and localization [10–13]. It has been suggested that PLA_2_ isoforms are involved either in the promotion or in the resolution of inflammation depending upon cell type and generation of eicosanoid [14–16]. In this context cytosolic phospholipase A_2_s (cPLA_2_s) are the main enzymes mediating arachidonic acid release and pro-inflammatory eicosanoids production. Moreover these biomolecules are associated with chronic inflammation which is a recognized risk factor for carcinogenesis [17–19]. The overall aim of this study was to investigate the involvement of particular cPLA_2_ which could be proposed as future therapeutic target during cigarette smoke induced pathologies in alveolar epithelium and results are reported in present paper.

## Methods

### Materials

WI26 (type-I) lung epithelial cell line was procured from American type culture collection (ATCC), Rockville, MD (USA), (ATCC® CCL-95.1™, https://www.atcc.org/Products/All/CCL-95.1.aspx). A549 (type-II) lung epithelial cell line was procured from National centre for cell science (NCCS), pune, India (http://www.nccs.res.in/CR5.html). PLA_2_-inhibitors bromoenol lactone (BEL), arachidonyl trifluroethyl ketone (ATK), bromophenacyl bromide (BPB), DCFH-DA, FDA and other general reagents were purchased from Sigma (St. Louis, MO, U.S.A.). PLA_2_-inhibitor YM26734 was purchased from Tocris bioscience, Bristol, UK.

### Methods

#### Cell culturing

Human lung epithelial type I (WI26) and type II (A549) cells were maintained in continuous culture at 37 °C temperature and 5 % CO_2_ in RPMI-1640 medium with 10 % fetal bovine serum (FBS). After every 48 h, depleted medium from the culture flask was replaced with fresh medium. Experimental design is represented as schematic view (Additional file 1: Figure S1).

### Cigarette smoke condensate preparation

Cigarette smoke condensate was prepared in our own laboratory according to standardized method of Kaushik 2009 [20], from most commonly used cigarettes (Gold Flake with filter) by the Indian population [21]. In brief smoke from a burning cigarette was sucked into a flask containing acetone with the help of a vacuum pump. Three ways connecting glass joint was used for suction. The rate of airflow was regulated by a valve so that cigarette burns upto bud in approximately 6 min. Acetone was evaporated under vacuum/nitrogen gas and the residue i.e. CSC was used for the experiments. CSC was dissolved in DMSO, in such a way that the final concentration of DMSO in plates did not exceed 0.025 % in culture medium. Further, dilution of CSC stocks was done with sterile PBS.

### Cell viability assay

Effect of CSC on cell viability was evaluated by the 3-(4,5- dimethylthiazol-2-yl)-diphenyltetrazolium bromide (MTT) dye uptake method [22]. Briefly, 2 x 10^3^cells were seeded in 96-well plates and allowed to grow overnight. After 24 h of priming, cells were treated with different concentrations of CSC for 24 h. Before treatment; medium was replaced with fresh medium. Four h before the end of desired time interval, 20 μl of MTT solution (2.5 mg/ml) was added to each well. After 4 h, resulting formazan crystals were dissolved in 40 μl of lysis buffer. The developed color was read at 540 nm on ELISA reader. The relative viability was calculated as described earlier.

### Reactive oxygen species

Levels of intracellular ROS were measured by the shift in fluorescent intensity resulting from oxidation of DCFH-DA fluorescence dye by the method of Wan et al. [23]. In brief, cells (0.5 x 10^5^ cells/well) were seeded into 12 well culture plates and allowed to grow overnight. Cells were challenged with CSC for 24 h. Before completion of treatment, cells were incubated with 5 μM DCFH-DA fluorescence dye for 30 min. After completion of treatment, cells were washed, harvested and re-suspended in ice-chilled PBS and analyzed by flow cytometery (FACScan) and shift in fluorescent peak was represented in terms of mean fluorescent intensity (MFI).

### Cellular integrity by FDA uptake

Cellular injury was determined by the FDA and ethidium bromide staining method [24]. FDA is an indicator of membrane integrity and cytoplasmic esterase activity. So the cells with intact membranes fluoresce green and cells with damaged membranes fluoresce red. Cells after CSC treatment was incubated with 10 μM FDA and 25 μM of ethidium bromide and was visualized under fluorescent microscope.

### Superoxide dismutase activity

Superoxide dismutase activity was estimated as described earlier [25]. In brief 2 x 10^6^ cells were plated in 100 mm^2^ culture dishes and allowed to grow for 24 h. After 24 h of CSC treatment, cells were washed, harvested and centrifuged at 200 x g for 10 min at 4^0^C. Cell pellet was washed with ice-chilled PBS and resuspended in PBS. Cells were lysed by the three freeze-thaw cycles followed by sonication (Sonicator Q700, Qsonica) for 5 min in ice-chilled water. After centrifugation at 800 x g for 10 min at 4 °C, the supernatant was assayed for SOD.

### PLA_2_ assay

The effect of CSC on the PLA_2_ activity was measured in the presence/absence of PLA_2_-inhibitors by the modified method of price [26]. In brief, samples were freshly diluted in 2 mM HEPES at pH 7.5 and 20 μl of each sample mixture, or buffer mixture as negative control was put in the well of a round bottom 96 well micro plate. 180 μL of an assay mixture at pH 7.5 at 37 °C was added to each sample. The plate was immediately read at 600 nm at one minute intervals for five minutes. Immediately after addition of substrate, the blue color of dye turns yellow with pH change due to PLA_2_ activity and absorbance decreases.

### Reverse transcription-polymerase chain reaction (RT-PCR)

After treatment total RNA from both type of epithelial cells was isolated using the method of Chomcznski et al [27]. Analysis of mRNA levels of cPLA_2_ groups IVA, IVB and IVC was done by RT-PCR. In PCR reactions (25 μl) 100 ng cDNA was used as template DNA. The primer sequences for the expression of all the genes were taken from previously published literature [28]. The primer sequences used for this study are given in (Additional file 1: Table S1).

### Protein estimation

Protein content of cell lysate was measured in all samples by Bradford method [29]. Bovine Serum Albumin (BSA) was used as a protein standard.

### Statistical analysis

Values displayed in the results are mean ± standard deviation of at least three independent experiments carried out in triplicate. *P* < 0.05 was considered to be statically significant. One way analysis of variance (ANOVA) and *t* test were employed using SPSS software to govern statistical significance of the results.

## Results

### Effect of CSC on cell viability and reactive oxygen species in A549 and WI26 cells

**Fig. 1:**
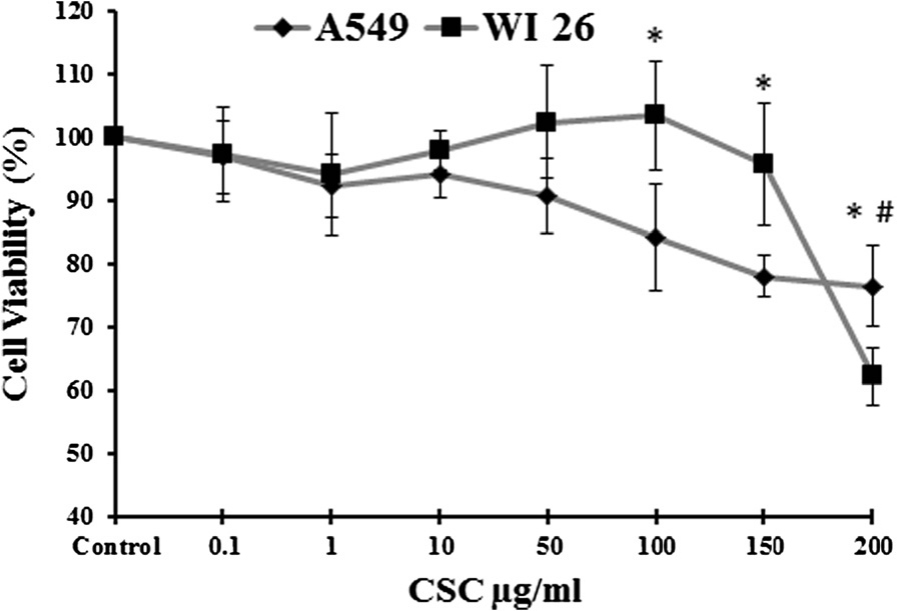
Effect of CSC treatment on cell viability (MTT assay) in A-549 and WI-26 cells. Both cell lines were treated with different concentrations of CSC for 24 h. The results are expressed as mean ± SD of three different experiments. ^*,#,^ *p* < 0.05. ^*^CSC compared with A549 control. ^#^CSC compared with WI26 control. Concentrations of CSC were in μg/ml

In A549 cells, it was found that cell survival decreased with increasing concentration of CSC from 1 μg/ml to 200 μg/ml and found to be 90.8 %, 84.2 %, 78.4 % and 76.4 % at 50 μg/ml, 100 μg/ml, 150 μg/ml and 200 μg/ml concentrations respectively. Whereas in WI26 cells, no significant change was observed in survival rate up to 100 μg/ml concentration of CSC. Moreover, it was observed that at 150 μg/ml and 200 μg/ml concentrations of CSC, survival rate of WI26 cells decreased to 95.7 % and 62.2 % (*p* < 0.05) respectively (Fig. 1).

**Fig. 2:**
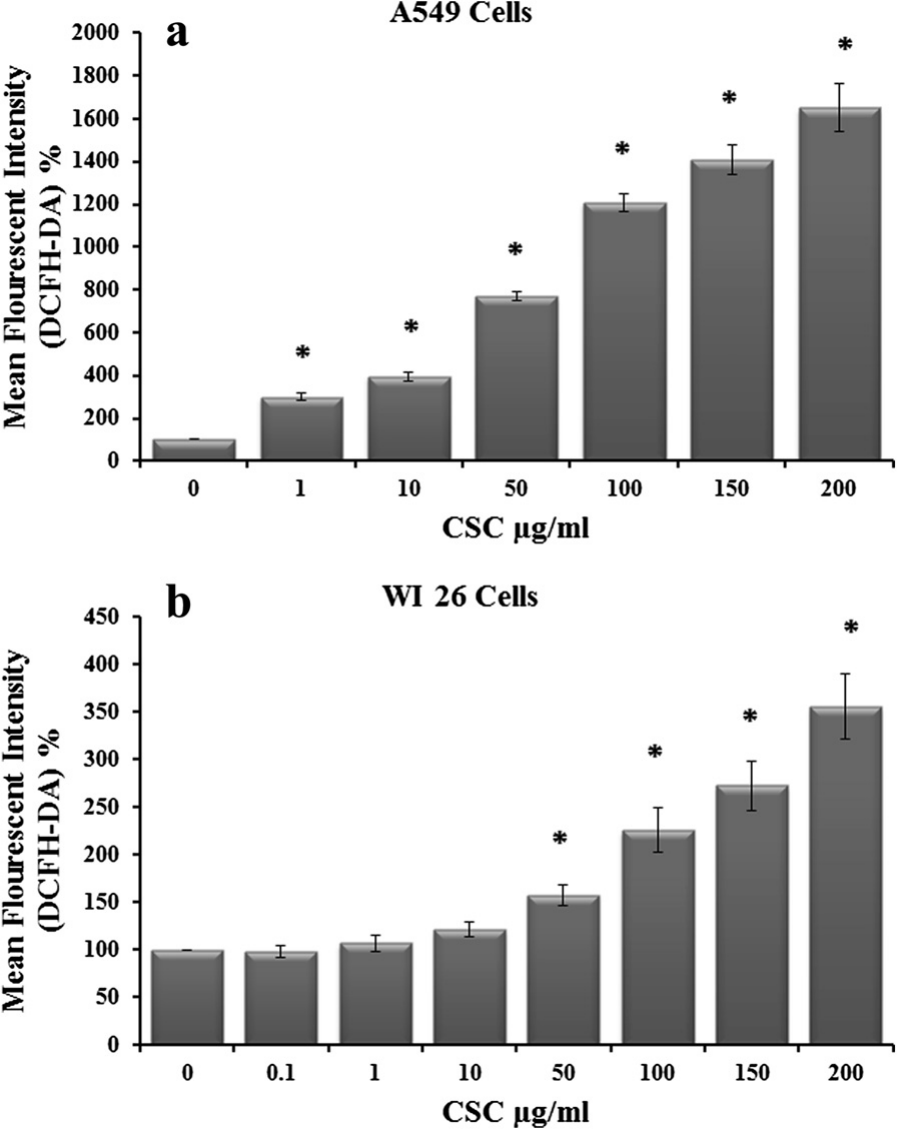
Effect of CSC treatment at different concentrations on ROS production in lung epithelial type II (A-549) cells (**a**) and lung epithelial type I (WI-26) cells (**b**), assayed by DCHF-DA fluorescent dye. The results are expressed as mean ± SD of three separate experiments. ^*^ *p* < 0.05. ^*^CSC compared with their respective control

Effects of CSC at various concentrations on ROS formation in the two cell lines are shown in Fig. 2. It was found that in A549 cells, even 1 μg CSC/ml increased the formation of ROS to 3 fold, which was further increased to 3.95 fold, 7.69 fold, 12.1 fold, 14.1 fold and 16.5 fold at 10 μg/ml, 50 μg/ml, 100 μg/ml, 150 μg/ml and 200 μg/ml concentrations respectively (Fig. 2a). In contrast to A549 cells, in WI26 cells CSC treatment increased the ROS formation to 1.21 fold, 1.58 fold, 2.26 fold, 2.73 fold and 3.56 fold at 10 μg/ml, 50 μg/ml, 100 μg/ml, 150 μg/ml and 200 μg/ml concentrations (Fig. 2b).

### Cells morphology

Since the morphology of two types of cells used in the present study were highly affected (Additional file 1: Figure S2) at higher concentrations (150 μg/ml and 200 μg/ml), these concentrations were not used in the next part of the study. Among all other lower concentrations, 50 μg/ml and 100 μg/ml of CSC induced maximum ROS production with optimum cell viability and hence were preferred in most of the other experiments. Time period was selected on the basis of our previous studies and preliminary experiments at different time intervals using ROS measurement and cell survival assay. 24 h time was found to be optimum and was used for current study.

### Effect of CSC on cellular integrity in A549 and WI26 cells

**Fig. 3:**
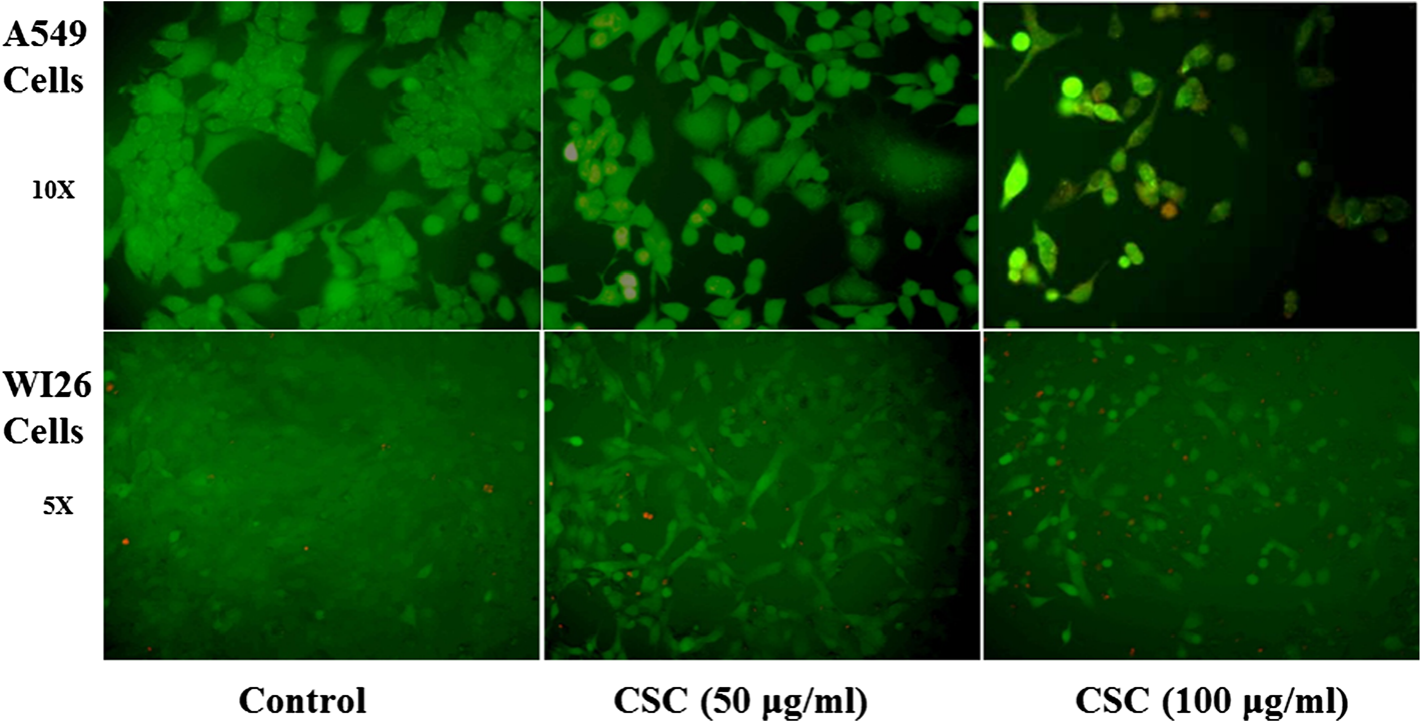
Effect of CSC on cellular integrity in A-549 and WI-26 cells using FDA and EtBr uptake assay

FDA uptake assay is an indicator of membrane integrity and cytoplasmic esterase activity. Figure 3 is showing the results of FDA and EtBr uptake in both types of cell lines. There was maximum uptake of FDA with no cellular accumulation of EtBr at control levels in both the cell lines. Accumulation of the fluorescein decreased and uptake of the EtBr increased in a concentration dependent manner in both the cell lines. Result obtained from the assay indicted the membrane integrity was stable at 50 and 100 μg/ml of CSC treatment.

### SOD levels in type I and type II epithelial cells

**Fig. 4:**
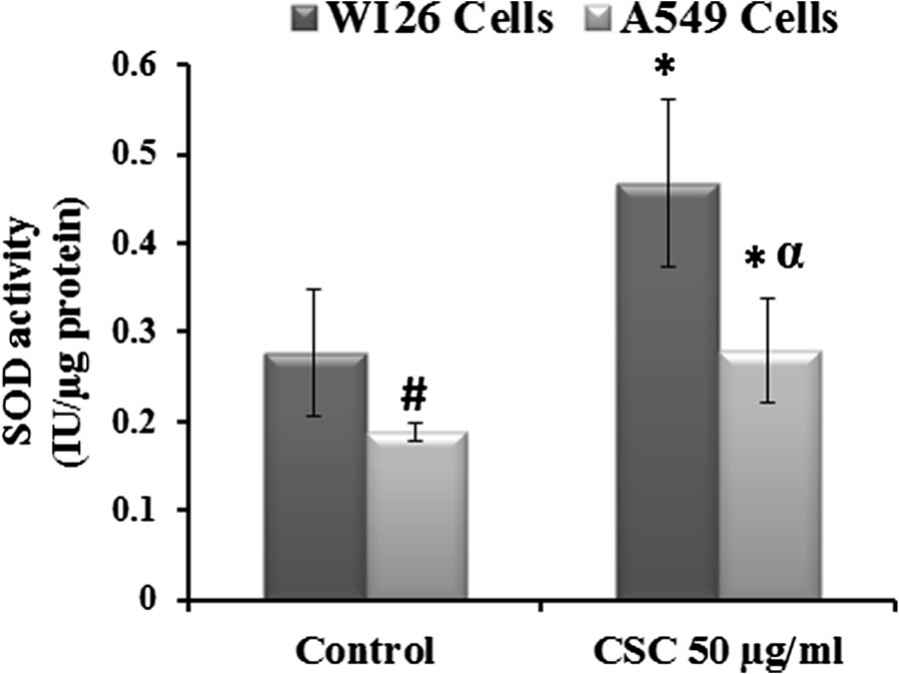
SOD activity after 50 μg/ml of CSC concentration at 24 h of treatment in A549 and WI26 cells. The results are expressed as mean ± SD of three separate experiments. ^*,#,α^ *p* < 0.05. ^*^CSC compared with their respective control, ^#^A549 control compared with WI26 control and ^α^compared with CSC threated WI26 cells

Effect of CSC treatment for 24 h on SOD in A549 and WI26 cells is shown in the Fig. 4. It was found that SOD activity was significantly higher in WI26 cells (0.278 IU/μg protein) as compared to A549 (0.188 IU/μg protein) cells. In the presence of 50 μg/ml of CSC concentration, SOD activity increased significantly in WI26 and A549 cells by 1.68 and 1.49 fold respectively (*p* < 0.05).

### CSC enhanced cPLA_2_ activity

**Fig. 5:**
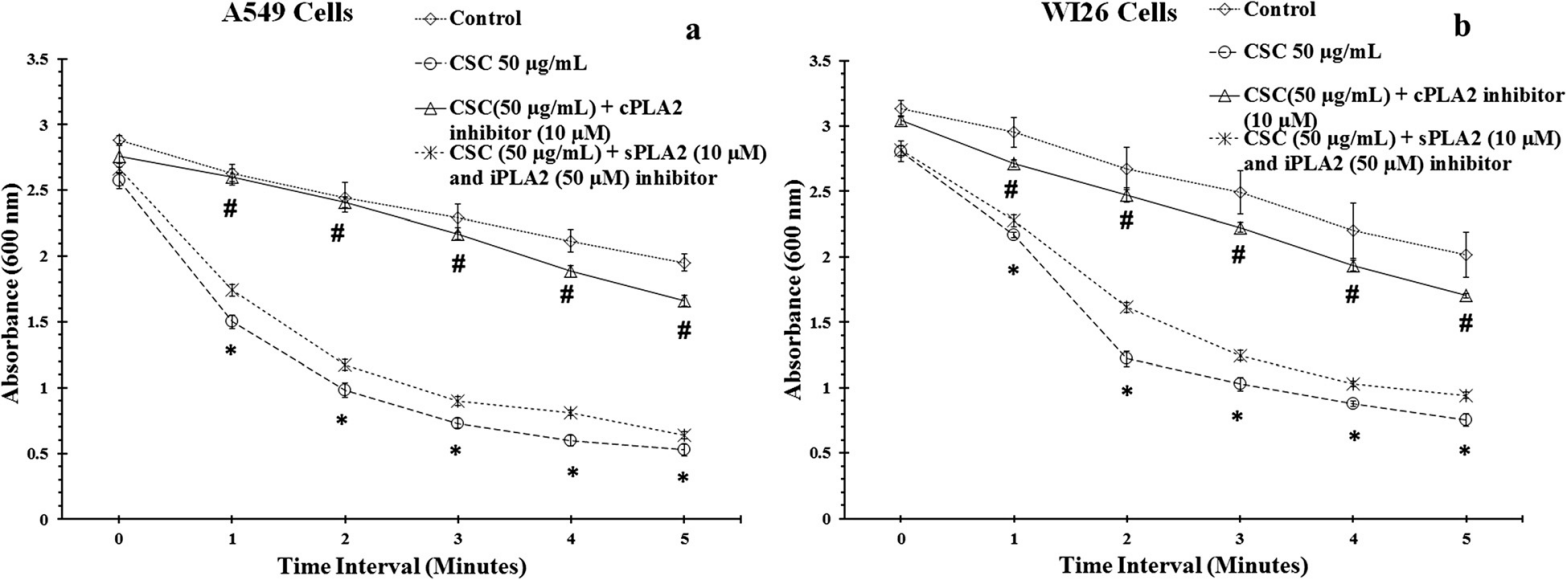
PLA_2_ activity at 50 μg/ml of CSC concentration alone or in combinations with PLA_2_ isoforms specific inhibitors at 24 h of treatment time in A549 cells (**a**) and WI26 cells (**b**). The decrease in absorbance is directly proportional to PLA_2_ activity. The results are expressed as mean ± SD of three separate experiments. *,^#^p < 0.05. *CSC 50 μg/ml activity response compared with their respective control at every minutes interval. #CSC 50 μg/ml in combination with cPLA_2_ specific inhibitor (ATK) activity response compared with their respective CSC 50 μg/ml alone at every minutes interval

Effect of CSC induced PLA_2_ activity in presence/absence of PLA_2_ inhibitors is shown in Fig. 5. Working PLA_2_inhibitors concentration was decided on the basis of cell viability, ROS and apoptosis experiments in the present study (Data not shown). At basal level we observed very low PLA_2_ activity in both type of lineages as optical density (OD) decreased at every minute interval from 2.88 to 2.63, 2.44, 2.29 2.11 and 1.95 in A549 cells (Fig. 5a), whereas from 3.13 to 2.95, 2.67, 2.49, 2.2 and 2.01 in WI26 cells. CSC at 50 μg/ml, significantly induced PLA_2_ activity in both type of cells as OD decreased at every minute interval from 2.57 to 1.5, 0.98, 0.73, 0.59 and 0.52 in A549 cells whereas from 2.80 to 2.17, 1.22, 1.02, 0.88 and 0.75 in WI26 cells (*p* < 0.05) (Fig. 5b). In presence of cPLA_2_ specific inhibitor ATK, we observed low CSC-induced PLA_2_ activity when compared to sPLA_2_ (YM26734) and iPLA_2_ (BEL) specific inhibitors in both type of cells. In presence of cPLA_2_ and sPLA_2_ + iPLA_2_ inhibitor, the decrease in OD was from 2.76 to 2.6, 2.41, 2.17, 1.88 and 1.6 (*p* < 0.05) and from 2.66 to 1.74, 1.17, 0.9, 0.80 and 0.63 respectively in A549 cells; whereas in WI26, OD decreased from 3.04 to 2.71, 2.47, 2.22, 1.93 and 1.70 (*p* < 0.05) and from 2.81 to 2.27, 1.61, 1.24, 1.02 and 0.94 respectively.

**Fig. 6:**
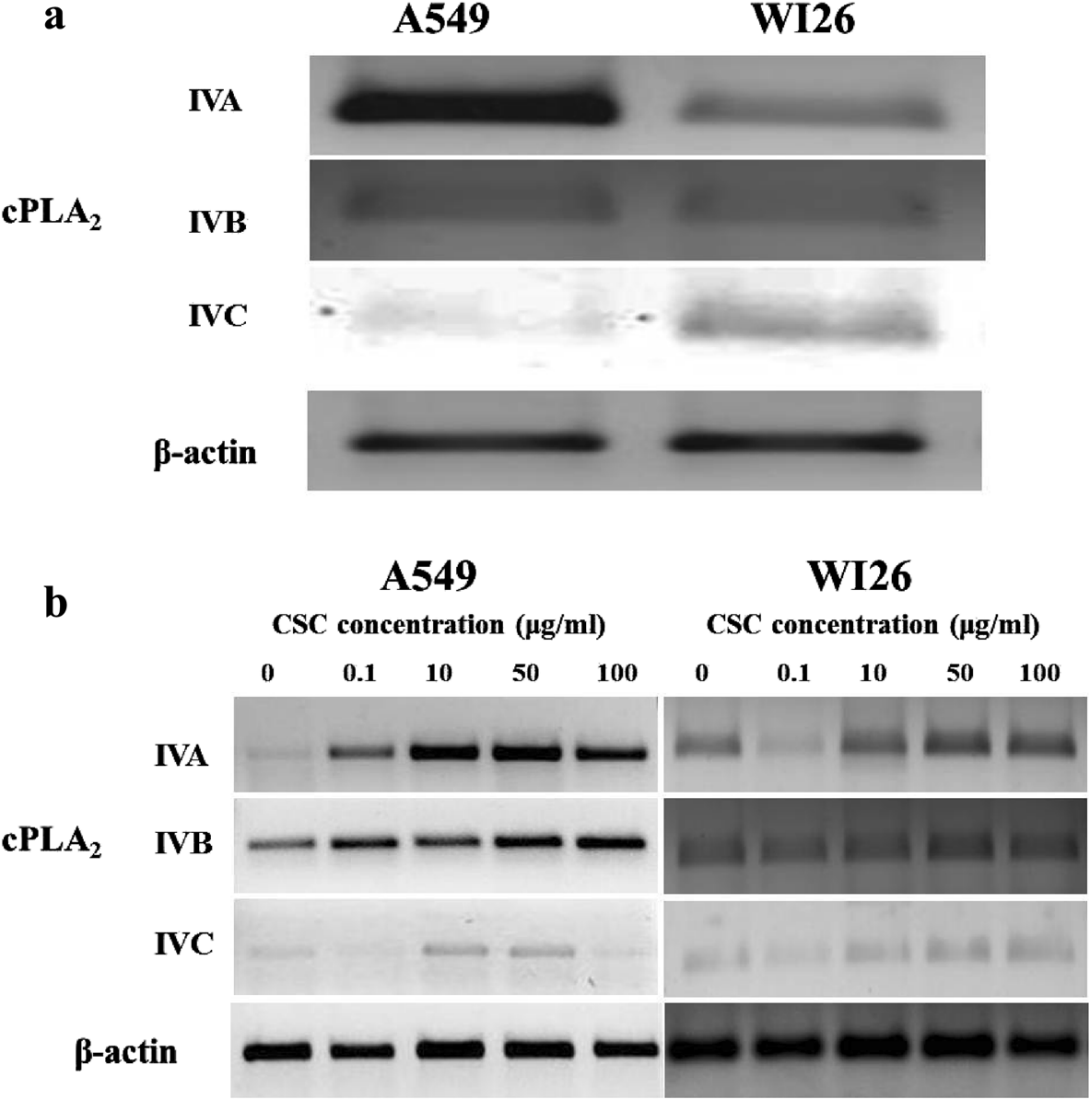
mRNA expression of different cPLA_2_ groups at constitutive level (**a**) and in presence of different concentrations of CSC (**b**) in A549 and WI26 cells. β-actin was used as an internal control for normalization of mRNA levels of cPLA_2_ groups

### CSC enhanced cPLA_2_s mRNA expression

Transcriptional modulations of various selected PLA_2_ groups (IVA, IVB and IVC) were studied at basal level (Fig. 6a) and in presence of CSC as shown in Fig. 6b. Densitometric analysis of all cPLA_2_ groups was done (Table S2). In A549 cells, mRNA expression of all the cPLA_2_ groups increased significantly in concentration dependent manner in all CSC treated cells and the most affected group was IVA (3.98 fold) followed by IVC (2.97 fold) and IVB (1.62 fold) (*p* < 0.05). In WI26 cells also, CSC treatment showed significant increase in mRNA expression of cPLA_2_ groups in a concentration dependent manner and the most prominently induced PLA_2_ group was IVA (1.95) followed by IVB (1.31) and IVC (1.98) (*p* < 0.05).

## Discussion

The lung is the only organ in the entire human architecture which has the greatest exposure to atmospheric oxygen and other environmental toxicants such as cigarette smoke [30, 31]. As we all knew that cigarette smoke is a highly complex mixture having more than 5000 chemicals including high concentration of free radicals and many of its components are known to be carcinogens, co-carcinogens and mutagens [5]. Among all type of cigarettes available in the Indian market, one of the most commonly consumed includes Gold Flake (with filter) [21] and same was used in the current study.

Cigarette smoke-induced chronic inflammation leads to the destruction of alveolar septae, resulted to the loss of elasticity and surface area for gas exchange, known as emphysema [9] and rapidly induces production of ROS thereby impairing endothelial functions [32]. The mechanisms leading to these changes after exposure of cigarette smoke were not yet completely understood in the two types of alveolar epithelial cells. However increased level of free radicals/oxidants in epithelial cells due to cigarette smoke leads to oxidative stress which is considered to be one of the major factors responsible for cell damage and death [31, 33, 34]. Using two lineages of epithelial cell lines, we showed that exposure of CSC leads to decrease in cell viability, increase in ROS production, loosed membrane integrity and altered morphology in a dose dependent manner at 24 h time period. All these observed parameters are well known hallmarks of inflammation and oxidative stress. Optimum time period and different doses of CSC were already reported by our group’s previously and same has been used in the present study [20]. In the continuation of previous study, we observed that CSC-induced ROS formation was found to be increased in concentration dependent manner in both WI26 (type I) cells as well as in A549 (type II) cells. However, the ROS formation was higher in A549 cells in comparison of WI26 cells. One of the possible reasons of such difference seems to be due to the difference in antioxidant status in two types of cells. To find out the reason behind, we investigated SOD levels in both type of cells and the results obtained from the study clearly indicate that WI26 cells had higher SOD activity in comparison of A549 cells alone and in combination with CSC treatment, its justify difference of ROS level finding among two types of lineages. Our finding shows that type II (A549) cells are comparatively more sensitive than type I (WI26) cells in terms of cell viability, higher ROS production as well as lower SOD levels. Moreover, type-I cells are highly exposed to airway insults and have been described as terminally differentiated cells, suggesting that they are incapable of cell division and cannot change their phenotypic expression. On the other hand, type-II epithelial cells are considered as progenitor cells which proliferate to re-epithelialize the damaged alveolar surfaces and then they transforms into the type-I epithelial cells. In these distinctive circumstances, type-II epithelial cells may be considered to show characteristics of stem cells mechanisms in alveolar repair [35–37] and seems to be very sensitive in contrast to type-I epithelial cells as we observed in the present study.

PLA_2_s are the important molecules involved in the remodeling of the membrane lipids and also in modulation of cell signaling which contributes to either the promotion or the resolution of inflammation during various lung pathologies [38, 39]. Moreover PLA_2_ activity in cancer tissues found to be much higher than normal cells [40]. In this direction, we examined the PLA_2_ activity in both type of lineages. In our observations, we found increased PLA_2_ activity after exposure of CSC. Our results are in correlation with another recently described study in which increased PLA_2_ activity has been reported after cigarette smoke exposure [41]. PLA_2_s have various isoforms but cPLA_2_s seems to play crucial role in CSC-induced inflammatory conditions. Results obtained from the present study indicated maximum decrease in cPLA_2_s activity in both type of cells by using commercially available isoform specific PLA_2_ inhibitor(s). It is well documented that cPLA_2_s have key regulatory roles in the invasive migration, proliferation, and capillary-like tubule formation of vascular endothelial cells as well as in tumor angiogenesis in lung cancer (mouse models) [42]. Our study is well supported by another recent research in which involvement of cPLA_2_ has been reported in asthmatic and COPD cases [43]. Moreover, several cPLA_2_ isoform specific inhibitors are commercially available and are in clinical trials for various inflammatory diseases and has been discussed in the review by Victoria [44]. The results of CSC simulated lung epithelial cell lineage treated with cPLA_2_ specific inhibitor such as ATK may also support the role of cPLA_2_ in lung pathologies.

Furthermore, to find out the role of specific cPLA_2_, we also characterized the comparative transcriptional level expression of cPLA_2_s at constitutive level and it was observed that all cPLA_2_s expressed in human lung epithelial type I (WI26) and type II (A549) cells. In presence of cigarette smoke condensate there was an increase in all cPLA_2_ groups but most predominantly induced group was PLA_2_ IVA in two different lineages of cells. In best of our knowledge, we are first group to report maximum expression of PLA_2_ IVA among all cPLA_2_s. The involvement of PLA_2_ IVA is already documented in several diseases and it has been used as a target molecule [45]. In this context, targeting group IVA may built-up new prospects in CSC-induced lung pathologies.

CSC-induced difference in the expression level of cPLA_2_s seems to play crucial role during lung pathologies. In this prospective, we also observed that CSC-induced cPLA_2_s expression and ROS levels were comparatively higher in A549 cells when compared to WI26 cells. Our finding clearly advocates that CSC-induced expression level of cPLA_2_ group’s seemed to be dependent on ROS levels generated. Our observation is supported by another study which suggested that cigarette smoke activates cPLA_2_expression through NADPH oxidase/ROS in human tracheal smooth muscle cells [8].

The role of specific cPLA_2_s in cigarette smoking induced lung pathologies remains elusive due to combine effect of cigarette smoke constituents, smaller to larger extent expression of all isoforms and distinct functions attributable to each isoform. Still PLA_2_ IVA had maximum expression in both lineages and can be of maximum interest in CSC induced inflammatory diseases. Now a days combine strategies are required to deal with such inflammatory situations. Firstly, dietary intake of biological molecules which are rich source of antioxidants and have anti-inflammatory effects [46]. Secondly, along with healthy diet use of cPLA_2_s specific analogs especially for PLA_2_ IVA may be a novel effective therapy in cigarette smoke related lung pathologies.

## Conclusion

In conclusion, it is worth mentioning; here the present study indicates involvement of specific cPLA_2_ IVA as a potential biomolecule candidate during cigarette smoke induced oxidative stress in type I and type II alveolar epithelial cells and strategies to target them may be key approach in cigarette smoke induced lung pathologies.

## Additional file

Additional file 1: Supplementary data. **Table S1.** Primer Sequences of various genes. **Table S2.** CSC-induced mRNA expression of various cPLA_2_ groups in A-549 and WI-26 Cells. The values (mean ± SD of three different experiments) are in folds of control values. **Figure S1.** Schematic presentation of proposed plan of experimental design. **Figure S2.** Effect of CSC treatment at concentrations’ of 150 μg/ml (b) and 200 μg/ml (c) on cell morphology in lung epithelial type II (A-549) cells and type I (WI-26) cells. Figure 2a shows the normal morphology in two types of the cells (Magnification 5x). (DOCX 1098 kb)
