# Supplementary material for: Cytosolic phospholipase A2 (cPLA2) IVA as a potential signature molecule in cigarette smoke condensate induced pathologies in alveolar epithelial lineages
Source: Lipids Health Dis. 2016 Aug 15;15:129. doi: 10.1186/s12944-016-0300-x (PMC4986351; doi:10.1186/s12944-016-0300-x)
Supplement: Additional file 1: — Supplementary data. Table S1. Primer Sequences of various genes. Table S2. CSC-induced mRNA expression of various cPLA2 groups in A-549 and WI-26 Cells. The values (mean ± SD of three different experiments) are in folds of control values. Figure S1. Schematic presentation of proposed plan of experimental design. Figure S2. Effect of CSC treatment at concentrations’ of 150 μg/ml (b) and 200 μg/ml (c) on cell morphology in lung epithelial type II (A-549) cells and type I (WI-26) cells. Figure 2a shows the normal morphology in two types of the cells (Magnification 5x). (DOCX 1098 kb) [file 12944_2016_300_MOESM1_ESM.docx]

**Supplementary Data**

ENGROSSMENT OF CYTOSOLIC PHOSPHOLIPASE A_2_ GROUPS IN CIGARETTE SMOKE INDUCED LUNG PATHOLOGIES: IN-VITRO

Subodh K Yadav^a^, Sanjeev K Sharma^a^, Abdullah Farooque^b^, Gaurav Kaushik^a,c^, Balwinder Kaur^a^, Chander Mohan Pathak^a^, BS Dwarakanath^b,d^, Krishan Lal Khanduja^a *^

^a^Department of Biophysics, PGIMER Chandigarh, India-160012

^b^Institute of Nuclear Medicine and Allied Sciences, New Delhi, India.

^c^Present address: Department of Surgery, School of Medicine, KU Medical Center (KUMC), Kansas City, KS-66160, USA

^d^Present address: Central Research Facility, Sri Ramachandra University, Porur, Chennai 600116, India

**Table S 1**

Primer Sequences of various genes

| **Gene** | **Primer Sequence** | **Product Size (bp)** |
| --- | --- | --- |
| **PLA_2_-IVA** | [s], 5’-GTTGCTGGTCTTTCTGGCTC-3’ -20 mer [a], 5’-GGTAAAGGGCATTGTGCAGT-3’ -20 mer | 313 |
| **PLA_2_-IVB** | [s], 5’-GAGCTTCGTGCTGGATGTG-3’ -19 mer [a], 5’-CGGATGGAACAGGAAATGC-3’ -19 mer | 273 |
| **PLA_2_-IVC** | [s], 5’-CGATTTACCCGAGGAGTGG-3’ -19 mer [a], 5’-GCTTCCGAAGTGGGTTATGG-3’ -20 mer | 329 |
| **β-Actin** | [s], 5’- TCTACAATGAGCTGCGTG -3’ -18 mer [a], 5’- CCTTAATGTCACGCACGA-3’ -18 mer | 372 |

**Table S 2**

CSC-induced mRNA expression of various cPLA_2_ groups in A-549 and WI-26 Cells. The values (mean ± SD of three different experiments) are in folds of control values.

| **cPLA_2_ groups** | **A-549** | | | | | **WI-26** | | | | |
| --- | --- | --- | --- | --- | --- | --- | --- | --- | --- | --- |
|  | **CSC Concentration (μg/ml)** | | | | | **CSC Concentration (μg/ml)** | | | | |
|  | **0** | **0.1** | **10** | **50** | **100** | **0** | **0.1** | **10** | **50** | **100** |
| **IVA** | 1 | 2.41±0.10^*^ | 3.64±0.37^*^ | 3.98±0.31^*^ | 3.86±0.33^*^ | 1 | 1.19±0.06^*^ | 1.42±0.11^*^ | 1.61±0.06^*^ | 1.95±0.08^*^ |
| **IVB** | 1 | 1.29±0.03^*^ | 1.38±0.03^*^ | 1.54±0.1^*^ | 1.62±0.11^*^ | 1 | 1.02±0.05 | 1.18±0.05^*^ | 1.28±0.06^*^ | 1.31±0.06^*^ |
| **IVC** | 1 | 1.24±0.08 | 1.48±0.10^*^ | 2.68±0.26^*^ | 2.97±0.27^*^ | 1 | 0.64±0.05^*^ | 1.16±0.01 | 1.51±0.11^*^ | 1.98±0.19^*^ |

*indicate p< 0.05. *CSC compared with their respective control


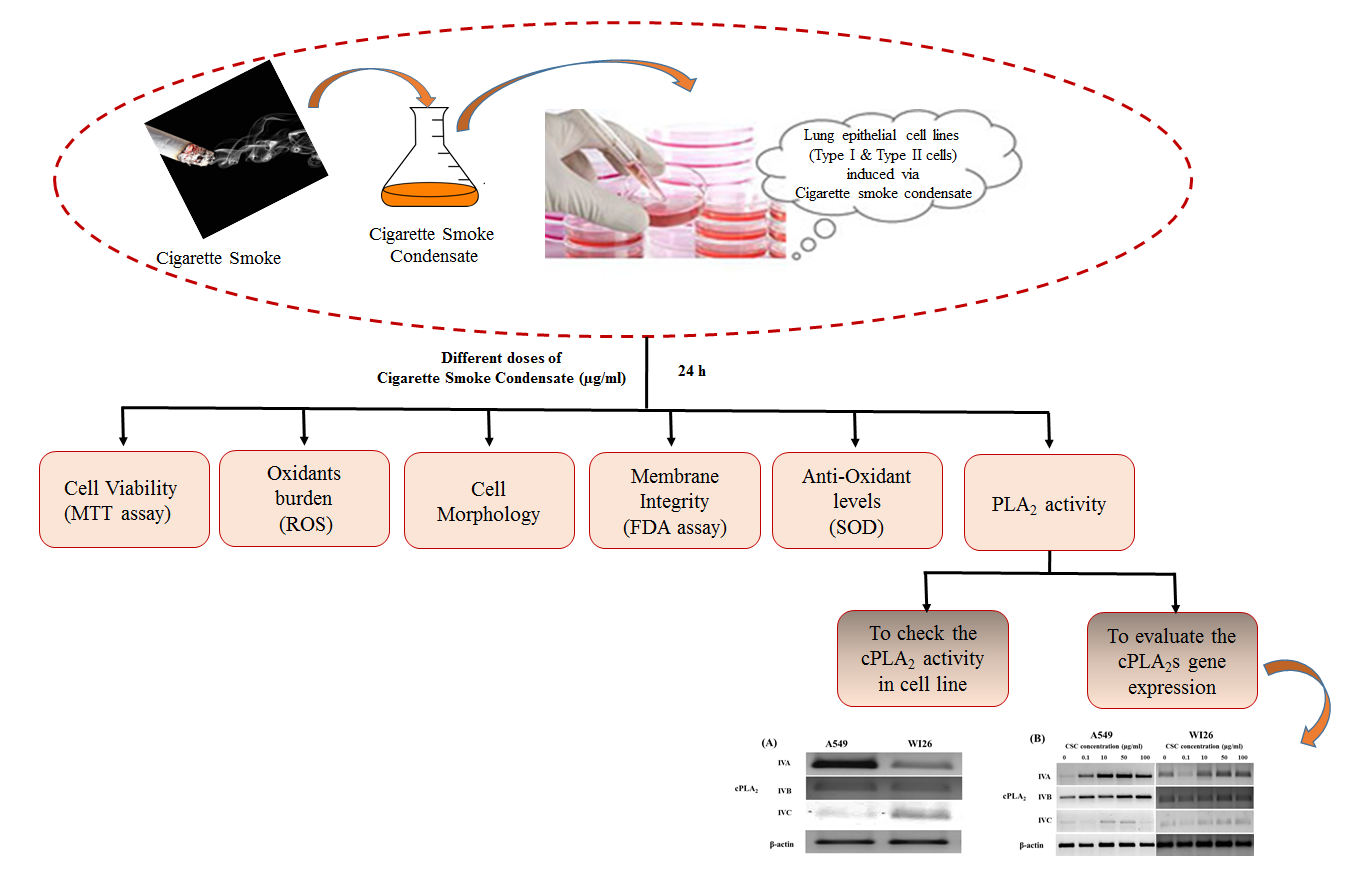


Figure S1:- Schematic presentation of proposed plan of experimental design


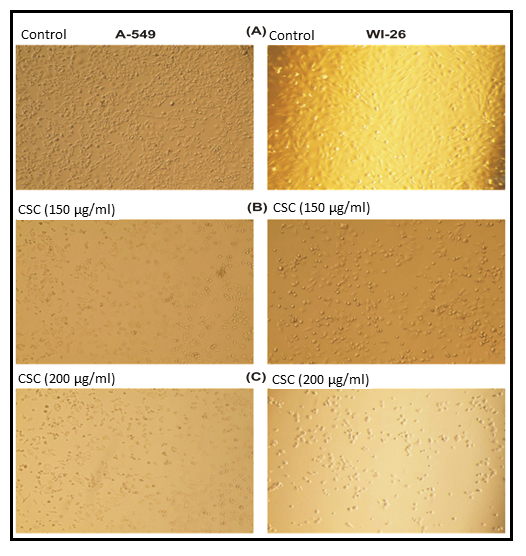


Figure S2 :- Effect of CSC treatment at concentrations` of 150 μg/ml (B) and 200 μg/ml (C) on cell morphology in lung epithelial type II (A-549) cells and type I (WI-26) cells. Figure 2A shows the normal morphology in two types of the cells (Magnification 5x).
